# Cost-effectiveness of recommended nurse staffing levels for short-stay skilled nursing facility patients

**DOI:** 10.1186/1472-6963-5-35

**Published:** 2005-05-10

**Authors:** David A Ganz, Sandra F Simmons, John F Schnelle

**Affiliations:** 1 Robert Wood Johnson Clinical Scholars Program, Veterans Affairs Greater Los Angeles Health Care System and University of California, Los Angeles, 911 Broxton Plaza, Los Angeles, CA 90024, USA; 2 Borun Center for Gerontological Research, University of California, Los Angeles and Jewish Home for the Aging, 7150 Tampa Avenue, Reseda, CA 91335, USA

## Abstract

**Background:**

Among patients in skilled nursing facilities for post-acute care, increased registered nurse, total licensed staff, and nurse assistant staffing is associated with a decreased rate of hospital transfer for selected diagnoses. However, the cost-effectiveness of increasing staffing to recommended levels is unknown.

**Methods:**

Using a Markov cohort simulation, we estimated the incremental cost-effectiveness of recommended staffing versus median staffing in patients admitted to skilled nursing facilities for post-acute care. The outcomes of interest were life expectancy, quality-adjusted life expectancy, and incremental cost-effectiveness.

**Results:**

The incremental cost-effectiveness of recommended staffing versus median staffing was $321,000 per discounted quality-adjusted life year gained. One-way sensitivity analyses demonstrated that the cost-effectiveness ratio was most sensitive to the likelihood of acute hospitalization from the nursing home. The cost-effectiveness ratio was also sensitive to the rapidity with which patients in the recommended staffing scenario recovered health-related quality of life as compared to the median staffing scenario. The cost-effectiveness ratio was not sensitive to other parameters.

**Conclusion:**

Adopting recommended nurse staffing for short-stay nursing home patients cannot be justified on the basis of decreased hospital transfer rates alone, except in facilities with high baseline hospital transfer rates. Increasing nurse staffing would be justified if health-related quality of life of nursing home patients improved substantially from greater nurse and nurse assistant presence.

## Background

Transfer back to the hospital is a common problem for patients admitted to skilled nursing facilities (SNF), with an estimated 18% of patients transferring to the hospital within the first 30 days of admission, and 38% within the first 90 days [1]. Avoiding hospital transfer in frail older individuals is desirable if care can be provided in the SNF because of the many adverse effects associated with hospitalization [2]. In addition, evidence suggests that SNF patients are often inappropriately hospitalized. One study which reviewed 100 unscheduled transfers to hospital found that 36% of transfers to the emergency room and 40% of hospital admissions from SNF were inappropriate, with poor quality of care in the SNF at least partially implicated [3]. Another study of processes and outcomes of care for Medicare SNF patients with acute heart failure found that if the patient's condition changed during the night shift, when staffing is generally lower, the odds of rehospitalization or emergency department evaluation were increased fourfold, suggesting an implicit connection between nurse staffing and rehospitalization rates among SNF patients [4].

This implicit relationship between nurse staffing and rates of hospital transfer was made explicit in the Center for Medicare and Medicaid Services (CMS) December 2001 report to Congress entitled "Appropriateness of Minimum Nurse Staffing Ratios in Nursing Homes [5]." This report argued that registered nurse, total licensed staff and nurse assistant staffing levels are related to rehospitalization rates in short-stay nursing home patients, and that certain staffing thresholds exist, below which quality problems are more likely. Since then, the Institute of Medicine, in a report entitled "Keeping Patients Safe: Transforming the Work Environment of Nurses," supported the adoption of the minimum staffing ratios found in the report to Congress [6]. In addition, in at least one instance, state legislators have proposed minimum nurse staffing levels consistent with those recommended in the CMS [7]. To test the hypothesis that reductions in hospital transfer rates would offset increased labor costs associated with higher staffing, we created a simulation model to gauge the costs and effects of recommended minimum staffing ratios for short-stay nursing home patients.

## Methods

### Model

We compared two scenarios for patients newly admitted to skilled nursing facilities from the hospital: 1) median staffing and 2) recommended staffing. The median staffing scenario used median staffing time for registered nurse (RN), licensed staff (RN plus licensed practical nurse (LPN)) and nurse assistant (NA) hours per patient day as defined in the CMS report to Congress. These median values per patient day were 2.02 hours for NAs, 1.02 hours for RNs plus LPNs, and 0.38 hours for RNs [5]. We used median staffing instead of mean staffing levels because the staffing data were highly skewed, and we considered the median staffing data more representative of most U.S. facilities.

The recommended staffing scenario used weighted average threshold values for staffing below which there was an increased likelihood of transfer to the hospital based on the CMS report to Congress. These recommended values were 2.37 hours for NAs, 1.14 hours for RNs plus LPNs, and 0.55 hours for RNs [5]. Under recommended care, NA, RN plus LPN, and RN staffing levels were higher than median values, while LPN hours were slightly lower.

**Figure 1:**
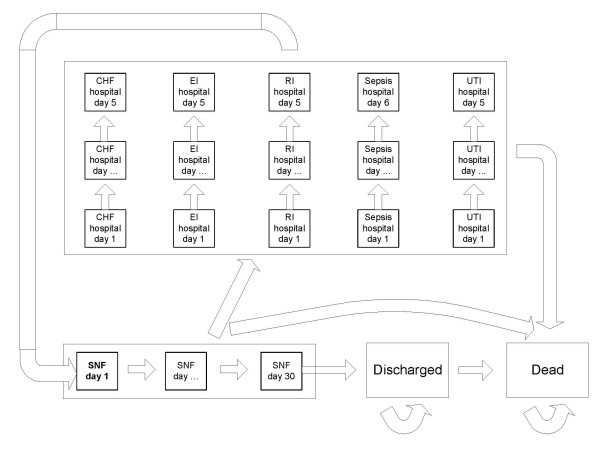
**Schematic diagram of Markov model. **The model has a cycle length of one day. All patients begin in the bolded state entitled "SNF day 1." From SNF day 1, patients can transition to a second SNF day, be hospitalized for any one of five conditions, or die. If patients spend 30 consecutive days in the SNF without being hospitalized, they transition to the "Discharged" state, where they remain until they die. If patients are hospitalized, they spend five or six days in the hospital, depending on the condition for which they are hospitalized, unless they die while in hospital. Upon completing their hospital course, patients then return to SNF day 1. Abbreviations: SNF, skilled nursing facility; CHF, congestive heart failure; EI, electrolyte imbalance; RI, respiratory infection; UTI, urinary tract infection.

We developed a Markov cohort simulation to track the costs and health outcomes for patients hypothetically assigned to one of the two staffing scenarios (median versus recommended). The Markov model was constructed using DATA Professional (TreeAge Software; Williamstown, MA). The model represents the typical flow of patients from their first day in the SNF to discharge, with a probability of being hospitalized for one of five conditions (congestive heart failure, electrolyte imbalance, respiratory infection, sepsis, and urinary tract infection) while in the SNF (Figure 1). These conditions were identified in the CMS report as being sensitive to licensed nurse and nurse assistant staffing (see section on transition probabilities). In the model, patients discharged from the hospital return to the SNF. Any patient completing 30 consecutive days in the SNF is then discharged from the SNF, which is not an overly restrictive assumption given an average length of stay of 22.9 days for SNF patients in the year 2000 [8]. Patients may also die at any point in the model, with probabilities of death that are higher while in hospital than while at the SNF or after discharge from the SNF. See Appendix A (in Additional File 1) for further details of model design.

### Transition probabilities

Transition probabilities for the Markov model are shown in Tables 1 and 2. The likelihood of being transferred to the hospital from the SNF for patients both under the median staffing and recommended staffing scenarios was based on Chapter 2 of the CMS Report to Congress on appropriateness of minimum nurse staffing ratios in nursing homes [5]. The authors of the report identified threshold values for the amount of NA, RN plus LPN, and RN hours per patient day below which there was an increased likelihood for facilities to be in the highest 10% of the sample for hospital transfer rates for the five conditions that were posited to be sensitive to licensed nurse and nurse assistant staffing mentioned previously. The analysis included all hospital transfers within 30 days of admission to the SNF that had one of these five conditions as the primary or secondary diagnosis. Logistic regression analyses were conducted with the object of determining the relationship between facility-level staffing (regardless of the patient mix within the facility) and the likelihood of being in the highest decile for hospital transfer rates. Odds ratios for a facility being in the highest decile for hospital transfer rates ranged from 1.31 for respiratory infection among facilities that fell below 1.05 licensed staff (RN plus LPN) hours per patient day to 2.43 for sepsis among facilities that fell below 2.40 NA hours per patient day [5]. Point estimates for these odds ratios were used for our base case analysis, with 95% confidence intervals used for sensitivity analysis, using the procedures discussed below.

**Table 1:** Parameters governing transition probabilities for the Markov model

**Parameter**	**Base Case**	**Biased toward recommended staffing**	**Biased against recommended staffing**	**Ref.**
30-day hospitalization rates from SNF*				
Congestive heart failure	3.9%	[7.8%]	[2.0%]	[5]
Electrolyte imbalance	4.4%	[8.8%]	[2.2%]	[5]
Respiratory infection	3.4%	[6.8%]	[1.7%]	[5]
Sepsis	1.4%	[2.8%]	[0.7%]	[5]
Urinary tract infection	3.0%	[6.0%]	[1.5%]	[5]
Case fatality rates at hospital*				
Congestive heart failure	1.2%	[2.5%]	[0.6%]	[9]
Electrolyte imbalance	1.7%	[3.4%]	[0.9%]	[9]
Respiratory infection	1.3%	[2.6%]	[0.7%]	[9]
Sepsis	3.1%	[6.1%]	[1.5%]	[9]
Urinary tract infection	1.5%	[3.0%]	[0.8%]	[9]
*Annual hazard of dying for SNF and discharged states*	30.3%	[15.2%]	[60.6%]	[10]

Parameters governing transition probabilities for the Markov model are listed here. Brackets refer to assumptions made for sensitivity analyses where data were not available. Base case values represent the best available estimate of the parameter in question. Values listed under the column "biased toward recommended staffing" and "biased against recommended staffing" represent extreme values of the parameter that are most favorable and least favorable to the recommended staffing scenario when tested in sensitivity analyses. Abbreviations: SNF, skilled nursing facility; Ref., reference.

*Probabilities of hospitalization for the five conditions mentioned were varied together in sensitivity analysis, as were case fatality rates.

**Table 2:** Relative risk reduction for hospitalization with recommended staffing

**Parameter**	**Base Case**	**Biased toward recommended staffing**	**Biased against recommended staffing**	**Ref.**
Nurse assistant factors*				
Congestive heart failure	3.4%	5.7%	0.2%	[5]
Electrolyte imbalance	3.2%	5.4%	0.2%	[5]
Sepsis	11.3%	14.5%	6.3%	[5]
Urinary tract infection	4.3%	7.2%	0.1%	[5]
Licensed staff factors*				
Electrolyte imbalance	2.9%	4.9%	0.4%	[5]
Respiratory infection	2.8%	5.0%	0.1%	[5]
Sepsis	6.1%	10.3%	0.4%	[5]
Urinary tract infection	4.7%	6.9%	1.8%	[5]
Registered nurse factors*				
Electrolyte imbalance	3.0%	5.2%	0.1%	[5]
Sepsis	5.7%	9.6%	0.4%	[5]
Urinary tract infection	3.9%	6.5%	0.4%	[5]

Parameters governing efficacy of recommended staffing levels at reducing hospital transfer rates are listed here. Not all staff were effective in preventing all five types of hospital conditions; only statistically significant reductions in hospital transfer were used in the analysis. Base case values represent the best available estimate of the parameter in question. Values listed under the column "biased toward recommended staffing" and "biased against recommended staffing" represent extreme values of the parameter that are most favorable and least favorable to the recommended staffing scenario when tested in sensitivity analyses, and were derived from 95 percent confidence intervals around efficacy parameters. Abbreviation: Ref., reference.

*All efficacy factors were varied simultaneously and also by group (RN, Licensed staff, and NA).

To convert these odds ratios into meaningful information for the Markov cohort simulation, we used additional data available in the CMS report to estimate total rates of hospital transfer for hypothetical facilities with median and recommended staffing. We assumed that the median-staffed facility had mean rates of hospital transfer for each of the five conditions as defined within the CMS report. Because the five conditions were not mutually exclusive, we downward-adjusted hospital transfer rates for each condition so that the rates for all five conditions totaled 16%, the overall 30-day hospital transfer rate for any diagnosis [5]. This assumes that 100% of hospital transfers had one of the five selected conditions as either the primary or secondary diagnosis, an assumption favorable to the recommended staffing scenario. In addition to assuming mean hospital transfer rates for the median-staffed facility, we also assumed an average risk of being in the highest decile of hospital transfer rates for any given condition (namely, 10%). Using this 10% probability together with the odds ratios of hospital transfer for each condition and nurse staffing type (NA, RN plus LPN, and RN), we were able to estimate the (reduced) likelihood of being in the highest 10% of hospital transfer rates for the facility with recommended staffing (and conversely, the increased likelihood of being in the lowest 90%).

Using percentile data on hospital transfer rates available in the CMS report [5], we were able to estimate bottom 90% and top 10% hospital transfer rates for each condition. Facilities with recommended staffing might have, for example, a 7% likelihood of being in the top 10% of hospital transfer rates, and therefore a 93% likelihood of being in the bottom 90%. Using these new weightings, we were able to estimate the relative reduction in hospital transfer rates for any of the five conditions (see Table 2) under the recommended staffing scenario. These values were then inserted into the Markov model. For the five staffing-sensitive conditions combined, we estimated an 8% relative risk reduction in 30-day hospital transfer rates (from 16% to 14.7%) under recommended staffing levels. We assumed that meeting recommended staffing for NA hours, licensed staff (RN plus LPN) hours, and RN hours were independent effects, because the CMS report does not indicate to what extent staffing levels are correlated [5]. For further details on efficacy calculations, see Appendix B (in Additional File 1).

Case fatality rates for hospitalized patients were taken from Medicare data [9]. While the actual case fatality rates for frail older SNF patients are likely higher than Medicare average rates, we did not have published estimates specifically for this population. Thus, we tested a range of fatality rates in sensitivity analyses. The likelihood of dying in the SNF or after discharge was based on a smoothed hazard from an eleven year follow up of nursing home patients in a single facility [10]. While this result may not be generalizable, it represents the longest published follow-up of patients likely to have similar levels of comorbidity as the target population. We tested a wide range of values (from 15.2% to 60.6% annually) for the hazard in sensitivity analyses given the uncertainty about this estimate.

### Health-related quality of life

The model measured health effects in terms of quality-adjusted life expectancy. The utility weights for the health states were derived from time-tradeoff scores collected from seriously ill patients (median age, 62.8 years) on day three of their hospital stay, and at two months post-hospitalization, as part of the SUPPORT study. These utility weights are shown in Table 3[11]. The mean time trade-off score for this seriously ill sample was 0.73 at day three of their hospital stay and 0.79 at month two post-hospitalization, where 0 represents death and 1 represents perfect health. This means that at day three of their hospital stay, on average patients accepted one year of life in their current state of health as being equivalent to 0.73 years in perfect health, whereas by month two after hospital discharge, patients considered one year of life in their current state of health as equivalent to 0.79 years in perfect health. While the improvement in health-related quality of life may appear small (0.73 to 0.79), it should be noted that these are average values and there was wide inter-individual variation in scores [11].

**Table 3:** Utilities, costs and discount rate

Parameter	**Base Case**	**Biased toward recommended staffing**	**Biased against recommended staffing**	**Ref.**
*Utilities*				
Hospital	0.73	0.41	[0.79]	[11]
Discharge	0.79	1.00	[0.73]	[11]
Days to reach discharge utility Under recommended staffing*	[30]	[15]	[30]	
*Hourly wages with benefits*				
Nurse assistant	$13.28	[$10]	[$20]	[13, 14]
Licensed practical nurse	$20.78	[$30]	[$15]	[13, 14]
Registered nurse	$32.94	[$20]	[$40]	[13, 14]
*Other nursing facility costs*	$193.5	[$400]	[$100]	[15]
*Hospitalization costs*				
Congestive heart failure**	$4603	[$9206]	[$2301]	[9]
Electrolyte imbalance**	$3913	[$7826]	[$1956]	[9]
Respiratory infection**	$4722	[$9444]	[$2361]	[9]
Sepsis**	$7142	[$14285]	[$3571]	[9]
Urinary tract infection**	$3853	[$7707]	[$1927]	[9]
*Daily cost for discharged patients*	[$0]	[$0]	$113	[17]
*Annual discount rate*	3%	0%	5%	[25]

All costs in 2002 U.S. dollars. Brackets refer to assumptions made for sensitivity analyses where data were not available or did not provide an adequate range for the parameter. Base case values represent the best available estimate of the parameter in question. Values listed under the column "biased toward recommended staffing" and "biased against recommended staffing" represent extreme values of the parameter that are most favorable and least favorable to the recommended staffing scenario when tested in sensitivity analyses.

*Number of days in skilled nursing facility required to go from utility at admission to discharge level of 0.79 under recommended staffing. Average staffed facilities were assumed to require 30 days to reach discharge utility level.

**Hospitalization costs were varied together. Hospitalization costs include Medicare reimbursement to the hospital plus initial and subsequent physician visits.

For the median-staffed scenario, we modeled the 30-day "post-acute" period as having a linear improvement in utility (health-related quality of life) from 0.73 on admission to 0.79 on discharge from the SNF. Preliminary analyses demonstrated that the cost-effectiveness ratio was sensitive to the differential in health-related quality of life between patients in facilities with median versus recommended staffing. Thus, we performed a sensitivity analysis in which health-related quality of life within the recommended-staffing scenario increased twice as rapidly in the nursing home as within the median-staffing scenario, with health-related quality of life leveling off at 0.79 by 15 days in the recommended-staffing scenario instead of at 30 days as in the median-staffing scenario. For further details on utilities, please see Appendix C (in Additional File 1).

### Costs

Our analysis adopts the cost perspective of Medicare, the typical payer for post-acute care. We report costs (shown in Table 3) in 2002 U.S. dollars. Where data were not available for the year 2002, prices were inflated to 2002 values using the medical care component of the Consumer Price Index [12]. We discounted all costs and health effects at 3% per year for the base case, with sensitivity analyses performed at discount rates of 0% and 5%. Costs were grouped into two categories, SNF costs and hospital costs. To obtain nursing costs in the SNF, hourly wages for RNs, LPNs, and NAs were estimated by ascertaining the wages of each staff type [13] and multiplying by a factor to account for fringe benefits, which represented 26.2% of total compensation for nursing home employees [14]. The adjusted hourly wages for each staff type were then multiplied by the hours worked by that staff type per patient day, and summed together, for each staffing scenario, creating a total cost of nursing care per patient-day. Non-nurse staffing related costs were estimated by taking the average daily reimbursement by Medicare to skilled nursing facilities and subtracting an estimated margin of 5%, and the nurse staffing costs calculated for the median-staffed facility [15]. Patients were assumed to have one visit by a physician on the initial day of their first admission to the SNF at a cost of $100 (CPT 99303) [16].

Hospital costs were estimated from the median Medicare reimbursement under Medicare Part A for each diagnosis [9]. Physician costs assumed an initial visit cost on day one (CPT 99223) and a subsequent visit cost (CPT 99233) on all days in which the patient remained in the hospital [16]. A range of total hospital costs (physician fees plus hospital costs), from half to double baseline values, was tested in the sensitivity analyses. We made no attempt to model costs after discharge from the SNF as this was not the focus of our analysis, so total daily costs per day after discharge were zero. However, we performed a sensitivity analysis on total daily costs per day for discharged patients to assess whether this would significantly affect our main findings. The upper limit of our sensitivity analysis on this parameter was $113/day, which reflects the inflation-adjusted average per-diem rate paid by Medicaid for long-term care [17].

## Results

### Base-case analysis

Under the base case assumptions, staffing SNFs at recommended levels resulted in discounted medical costs of $8941 per post-acute patient from admission to ultimate discharge, versus a cost of $8767 for facilities with median staffing (incremental cost $173.50 per patient). Discounted, quality-adjusted life expectancy was 2.28117 quality-adjusted life years (QALYs) in the facility with recommended staffing and 2.28063 QALYs in the median-staffed facility, for a difference of 0.00054 QALYs. The incremental cost-effectiveness ratio was $321,000 per discounted QALY gained.

Without quality adjustment, the cost-effectiveness ratio was $271,000 per discounted year of life saved, and $250,000 per undiscounted year of life saved. Undiscounted life expectancy was 3.13587 years in the recommended-staffing scenario and 3.13517 years in the median-staffing scenario, for a gain of 0.0007 life years per patient. Undiscounted costs were $8780 for the median-staffing scenario and $8953 for the recommended-staffing scenario, with an incremental cost of $173.60.

### Sensitivity analysis

#### Factors affecting transitions between states

We report results of sensitivity analyses in Table 4. In one-way sensitivity analysis, the cost-effectiveness ratio was most sensitive to the rate of hospitalization for the five nursing-care-sensitive conditions: congestive heart failure, electrolyte imbalance, respiratory infection, sepsis, and urinary tract infection. When the hospitalization rates from the SNF for these conditions were simultaneously doubled from their base case values, then the cost-effectiveness ratio was $36,000 per QALY. If hospitalization rates were simultaneously half their base case values, the cost-effectiveness ratio was $896,000 per QALY. The cost-effectiveness ratio was not sensitive to changes in mortality rates for any of the health states.

**Table 4:** One-way sensitivity analyses on selected parameters, expressed as dollars per quality-adjusted life year gained

**Parameter**	**Range (Best, Worst)**	**Biased toward recommended staffing**	**Biased against recommended staffing**
*Efficacy of optimizing nurse staffing*	See table 2		
All staff		$124,000	$2,508,000
NA		$225,000	$556,000
Licensed staff (RN + LPN)		$230,000	$510,000
RN		$289,000	$369,000
*Transition probabilities*			
Hospitalization rate	(double, half)	$36,000	$896,000
In-hospital mortality rate	(double, half)	$150,000	$793,000
Annual mortality rate in SNF or when discharged	(15%, 61%)	$181,000	$760,000
*Utilities*			
of "hospital" state	(0.41, 0.79)	$239,000	$343,000
of "discharged" state	(1.00, 0.73)	$218,000	$371,000
Time to discharge utility in recommended staffing group	(15 d., 30 d.)	$94,000	$321,000
*Costs*			
Hospitalization cost	(double, half)	$192,000	$386,000
NA wage	($10, $20)	$256,000	$455,000
LPN wage	($30, $15)	$292,000	$339,000
RN wage	($20, $40)	$193,000	$391,000
Non-nursing costs in SNF	($400, $100)	$243,000	$356,000
Daily costs for discharged patients	($0, $113)	$321,000	$428,000
*Discount rate*	(0%, 5%)	$297,000	$337,000

All costs in 2002 U.S. dollars. The column labeled "range" refers to the best-case and worst-case values tested in sensitivity analyses. "Double" and "Half" refer to double and half the base case values, respectively. SNF, skilled nursing facility; NA, nurse assistant; LPN, licensed practical nurse; RN, registered nurse.

The cost-effectiveness ratio was somewhat sensitive to the efficacy of nursing staff as a whole. When all staff (nurse assistants and licensed staff) were assumed to maximally reduce the risk of hospitalization for the conditions that were sensitive to their input (which we accomplished by setting the risk reduction for hospitalization to the favorable end of the 95% confidence intervals) the cost-effectiveness ratio was $124,000 per QALY. When staff were assumed to be minimally effective under recommended staffing conditions (by using the least favorable end of the 95% confidence intervals for staff efficacy at reducing hospital transfer rates), the cost-effectiveness ratio was $2,508,000 per QALY. The cost-effectiveness ratio was not sensitive to the contributions of individual staffing type (NAs, LPNs plus RNs, or RNs), nor was it sensitive to varying the efficacy of recommended staffing at preventing hospitalizations for a particular condition (e.g. sepsis).

#### Health related quality-of-life and costs

The cost-effectiveness ratio was sensitive to the rate of improvement of quality of life while in the SNF in the recommended-staffing scenario. The median-staffed group was assumed to require the entire 30-day stay in the SNF to reach the discharge level of health-related quality of life. If the group with recommended staffing reached the discharge level of health-related quality of life by 15 days, half-way through the expected stay and twice as quickly as the median-staffed group, the cost-effectiveness ratio was $94,000 per QALY. If it took the entire 30 days to reach the discharge value for health-related quality of life for the recommended staffing group, then the cost-effectiveness ratio was the base case, or $321,000 per QALY. The cost-effectiveness ratio was not sensitive to the utility of being in the "hospital" or "discharged" states. The cost-effectiveness ratio was not sensitive to any of the tested costs nor any of the tested discount rates.

## Discussion

To our knowledge, this is the first attempt to compare the cost and effectiveness of two different nurse staffing scenarios using a cost-utility framework. Because there are no randomized data on the efficacy of increasing nurse staffing in nursing homes to the minimums identified in the CMS report [5] and recommended by the Institute of Medicine [6], modeling can serve as a means of testing various hypotheses about efficacy of staffing interventions on different outcomes. Our simulation modeling results indicate that preventing hospital transfers alone is unlikely to make the recommended minimum-staffing ratios cost-effective by conventional medical standards, unless increases in staffing to the recommended levels are targeted to facilities with high hospital transfer rates. This result occurred because staffing effects on hospitalizations, though statistically significant as reported in the CMS study, were small in magnitude (we estimated a reduction in 30-day hospital transfer rates from 16% to 14.7% based on the CMS data).

The cost-effectiveness ratio was sensitive to the rapidity of improvement in health-related quality of life in the SNF in the recommended staffing scenario compared to the median-staffing scenario, but only when the differential improvement was quite marked. However, the approach we used is likely to underestimate the true benefit of increased staffing on quality of life, since it does not capture non-health-related benefits, such as physical comfort, attentiveness of staff, and social interaction. In the long-term care arena, there is evidence that staffing affects non-health-related quality of life. Residents in homes staffed at the recommended minimum levels were more likely to be out of bed during the day, were more socially engaged, and were assisted more frequently with incontinence, repositioning, and eating assistance [18]. In addition, when asked, nursing home residents prefer more frequent help with basic activities of daily living than they would typically get under routine median staffing conditions [19]. Furthermore, families of nursing home residents placed a high financial value on the incontinence and exercise care activities that are associated with higher staffed homes, valuing these staffing-intensive activities more than private rooms, which are successfully marketed and expensive [20]. To what extent these findings, which come from the long-term care population, translate to patients in post-acute settings is not clear, but it seems plausible that the benefits of staffing to short-stay patients extend beyond preventing hospitalization for acute sickness episodes and also include non-medical aspects of care.

The cost-effectiveness ratio was sensitive to the rate of hospital transfer from the nursing home, ranging from $36,000 per QALY at two-fold the baseline rate, versus $896,000 per QALY at half the baseline rate. This result occurred due to the greater absolute risk reduction in hospital transfers in the simulation model when the overall rates of hospital transfer are higher, as well as the downstream effects of rehospitalization, which include a higher mortality rate while in-hospital and additional days in the SNF after discharge from the hospital. Thus, our results predict that facilities with the highest baseline hospital transfer rates stand to benefit the most from meeting recommended nurse staffing levels.

Compared to other medical interventions, the base case cost-effectiveness ratio of $321,000/QALY was relatively expensive. For example, equipping suitable nursing home residents with hip protectors increases quality-adjusted life expectancy while saving society money [21,22]. A practice-initiated quality improvement intervention to improve treatment for depression demonstrated cost-effectiveness ratios between $9000 and $36,000/QALY (1998 U.S. dollars) [23]. However, performing annual Papanicolaou smears, a common medical practice, cost >$1,600,000/QALY (1995 U.S. dollars) when compared to performing them every two years [24].

Our analysis has several limitations, the most noteworthy being the limitations of the data on which the analysis is based. The recommended staffing levels required to prevent increased rates of hospital transfer were estimated through a retrospective analysis of data collected for administrative purposes, and the exact thresholds were determined through a post-hoc, iterative process designed to isolate the recommended staffing levels for each staff type. All the limitations of retrospective data analysis, most notably the potential inability to adjust adequately for case mix, thus affect our best estimate of cost-effectiveness. Also, the CMS report estimates median and recommended nurse staffing at the facility level. Short-stay patients may occupy a different percentage of beds in each facility, and the actual intensity of staffing for those beds might vary systematically from the facility-wide estimate in different ways for facilities with median and recommended levels of staffing. To compensate for these limitations, we varied the efficacy parameters over their 95 percent confidence intervals simultaneously, thus testing a broad range of possibilities, and the results were comparable.

This study focused on the costs and clinical benefits of recommended staffing, and not the costs of a staffing mandate. Thus, it did not include the costs for policy enforcement. A federal mandate to nursing facilities to staff at certain recommended levels requires an apparatus for accurate collection of staffing data and a mechanism for enforcement, and even then will not ensure full compliance. Predicting the consequences of a mandate was beyond the scope of our analysis, which focused on the immediate and downstream benefits of decreased hospital transfer rates under recommended staffing levels compared to median staffing levels.

We focused solely on patients recently discharged from the hospital, often known as "post-acute" patients, where the Medicare Part A program is typically the payer. These patients generally enter a SNF for a limited time to recuperate from their acute hospital stay and receive skilled therapies (e.g. physical therapy, occupational therapy, speech therapy, or intravenous antibiotics). This analysis did not attempt to model the effect of improving staffing for the "long-stay" residents, which constitute a different patient population [5], so our findings are only generalizable only to short-stay patients.

Strengths of the simulation model presented in this study include the clinical relevance of the model, which captures the situation faced by the post-acute care population. The model was quite robust to most tested variables, and plausibly sensitive to a few key variables.

## Conclusion

We conclude that an intervention to increase nurse staffing to recommended levels for short-stay patients would not be cost-effective on the basis of reduction in hospital transfer rates alone. Further research should quantify the non-health-related quality of life benefits experienced by short-stay patients as a function of recommended and median nurse staffing levels.

## Competing interests

The author(s) declare that they have no competing interests.

## Authors' contributions

DAG constructed the Markov model and drafted the manuscript. SFS and JFS contributed to the study design and critically revised the manuscript. All authors read and approved the final manuscript.

## Pre-publication history

The pre-publication history for this paper can be accessed here:



## Supplementary Material

Additional File 1 APPENDICES A-C TABLES 5-7 FOR MANUSCRIPT: Cost-effectiveness of recommended nurse staffing levels for short-stay skilled nursing facility patients. Click here for file
