# Supplementary material for: Cost-effectiveness of recommended nurse staffing levels for short-stay skilled nursing facility patients
Source: BMC Health Serv Res. 2005 May 10;5:35. doi: 10.1186/1472-6963-5-35 (PMC1145183; doi:10.1186/1472-6963-5-35)
Supplement: Additional File 1 — APPENDICES A-C TABLES 5-7 FOR MANUSCRIPT: Cost-effectiveness of recommended nurse staffing levels for short-stay skilled nursing facility patients. [file 1472-6963-5-35-S1.pdf]

APPENDICES A-C

TABLES 5-7

FOR MANUSCRIPT:

Cost-effectiveness of recommended nurse staffing levels for short-stay skilled nursing facility patients

David A Ganz,[1§] Sandra F Simmons,[2] John F Schnelle[2]

[1]Robert Wood Johnson Clinical Scholars Program, Veterans Affairs Greater Los Angeles Health Care System and University of California, Los Angeles, 911 Broxton Plaza, Los Angeles, CA 90024, USA
[2]Borun Center for Gerontological Research, University of California, Los Angeles and Jewish Home for the Aging, 7150 Tampa Avenue, Reseda, CA 91335, USA

[§]Corresponding author

E-mail addresses:

DAG: dganz@mednet.ucla.edu

SFS: ssimmons@ucla.edu

JFS: jschnell@ucla.edu

Appendix A: model design

The model is a Markov model with a one-day cycle length, and consists of 58 states:

- 30 skilled nursing facility (SNF) states, one for each day of residence in the SNF
- five states, each representing one day of a hospital stay for congestive heart failure
- five states, each representing one day of a hospital stay for electrolyte imbalance
- five states, each representing one day of a hospital stay for respiratory infection
- six states, each representing one day of a hospital stay for sepsis
- five states, each representing one day of a hospital stay for urinary tract infection
- one state for patients discharged from the SNF
- one "dead" state

The model runs for 3650 cycles (approximately 10 years) at which point over 95% of the cohort has died.

All patients begin in the SNF state called "SNF day 1." (see Figure 1 in main manuscript). This is meant to represent a group of patients just admitted to the skilled nursing facility after discharge from the hospital. The SNF states are a 30-stage tunnel, meaning that patients automatically progress from SNF day 1 to SNF day 2 to SNF day 3, etc., unless they die or are hospitalized. The tunnel is 30 stages in length to reflect the typical length of stay of patients in post-acute care as well as to mirror the outcome measure of interest, 30-day hospital transfer rates. While in the SNF states, patients accrue daily costs. These costs include nursing-related and non-nursing related costs. At inception, patients accrue a one-time cost for an initial physician visit. This cost is not accrued again on readmission to the skilled nursing facility after rehospitalization, since Medicare regulations stipulate that a physical exam on discharge from the hospital suffices for a patient readmitted to the nursing home within 30 days (Katherine

Ward, personal communication). While in the SNF states, patients may die without being rehospitalized, at the background rate used in the model for both "SNF" and "discharged" states [10].

After the 30th SNF state, patients are sent to the "discharged" state. Though the state is called "discharged," in real life some patients may continue to reside at the same facility for long-term care, while others may be sent home or to an assisted-living facility. Since the analysis does not concern itself with events beyond the time in the skilled nursing facility, this state is simply meant to capture accurately the remaining life expectancy of patients after residing in the skilled nursing facility. As such, patients in the "discharged" state remain in that state until they transition to the "dead" state, which they do at the background rate specified for both SNF states and the “discharged” state [10].

While patients reside in one of the SNF states, they may transition to any one of five hospital tunnels representing five different conditions: congestive heart failure, electrolyte imbalance, respiratory infection, sepsis, or urinary tract infection. Each of the hospital tunnels is a 5-state tunnel, with the exception of "sepsis," which is a 6-state tunnel. The length of the tunnels reflects the average length of stay for Medicare beneficiaries with the diagnosis-related group (DRG) that corresponds each of the five nursing-care sensitive conditions. For each state (day) in the hospital tunnel, patients accrue a cost for a daily physician visit as well as the daily cost of their hospitalization (the DRG payment to the hospital from Medicare divided by the length of the tunnel). On the first day, the physician visit charge is for an initial visit; on subsequent days, the charge is for a follow-up visit. While it is likely that patients in the hospital states would accrue additional costs (consultant physician bills, radiologist's bills for reading films, etc.), the diversity of actual care makes it impossible to produce a uniform estimate as to

what these additional costs would be. Instead, we vary hospital costs extensively in sensitivity analysis to see if they affect the conclusions of the analysis.

Appendix B: efficacy measures

The source data express hospital transfer rates from being below recommended staffing levels versus being at or above recommended staffing levels as an odds ratio of being in the decile of facilities with the highest hospital transfer rates. Since we did not have access to the source data, we had to convert the *odds ratio of being in the highest decile of hospital transfer rates* to a *relative risk of being hospitalized* for patients in facilities with recommended staffing compared to median staffing. This required three steps: 1) solving for the probability that a facility with recommended staffing would be in the highest decile of facilities for hospital transfer rates, 2) combining the probabilities of median-staffed facilities and facilities with recommended staffing of being in the decile of facilities with the highest transfer rates with the actual top 10% and bottom 90% hospital transfer rates to calculate a relative risk for hospitalization in general.

To solve for the probability that a facility with recommended staffing would be in the highest decile of facilities for hospital transfer rates, we made the assumption that a facility with median staffing levels, holding other variables constant, would have a ten percent probability of being in the decile of facilities with the highest hospital transfer rates. The basis for this assumption is that without knowing anything about the case-mix of a given facility, its likelihood of being in the highest 10% of facilities on any measure is 10%. Call this 10% probability p1. Now define p2 as the probability of a facility with recommended staffing levels being in the highest decile of hospital transfer rates. If we know p1 and we know the value of the odds ratio (p1/(1-p1))/(p2/(1-p2) for the comparison of being in the highest decile for facilities below and above recommended levels, it becomes clear that we can solve for p2. Odds ratios were taken

from Chapter 2, page 17 of the CMS report [5]. Results for p2 (point estimates and upper and lower confidence limits) are presented in Table 5.

We then need to reconstruct the top 10% and bottom 90% hospital transfer rates. In Chapter 2 of the CMS report, page 13 [5], a table lists hospital transfer rates at various percentiles of the facilities surveyed, as well as mean hospital transfer rates. The highest decile of hospital transfer rates can be reconstructed from the 90th and 99th percentile rates as a weighted average, namely 0.9*(90th percentile value) + 0.1*(99th percentile value). This produces a conservative (low) estimate of the highest decile of hospital transfer rates. Using mean hospital transfer rates and highest decile rates for all facilities, it becomes possible to derive an estimate of the "bottom 90%" transfer rate for all facilities, as follows: Mean rate=0.9*(bottom 90% transfer rate) + 0.1*(top decile transfer rate). Whereas the median-staffed facility is assumed to have the mean rate of hospital transfer, the facility with recommended staffing has the following transfer rate: (1-p2)*(bottom 90% transfer rate) + p2*(top decile transfer rate). A relative risk of hospital transfer can then be calculated.

Since hospital transfer diagnoses were not mutually exclusive, and the overall hospital transfer rate of 16% was significantly less than the sum of all the individual mean transfer rates, we downward adjusted mean transfer rates by a factor of 1.4625 so that the sum of all mean transfer rates was 16%. This same factor of 1.4625 was then used to adjust downward the 90th and 99th percentile transfer rates, and these adjusted values were used in the calculations described above. Calculations for the reductions in hospital transfer rates are presented in Tables 6 and 7.

Appendix C: quality of life weights

We chose to use patient preferences from the SUPPORT study [11] rather than community-based preferences for this analysis. The main reason was that the SUPPORT data more closely approximated the health scenario of our target population as compared with other available data. Specifically, the availability of a utility estimate for inpatients (which was likely to be a good surrogate for patients newly admitted to the skilled nursing facility) coupled with a follow-up measure using the same population and the same means of preference elicitation was highly attractive. The SUPPORT study also measured utilities at 6 months post-hospitalization; we did not use these because they were negligibly different than the 2-month values, and would have added unnecessary complexity to the model. Instead, we chose to focus on trying to model sensibly the recovery period between the inpatient stay and the first available follow-up measure (the 2-month follow-up utility).

Table 5. Solving for probability p2.

**NA < 2.37 HPPD**	**OR and 95% CI***	**p1**	**p2 and 95% CI**
Congestive Heart Failure	1.47 (1.02, 2.11)	0.10	0.070 (0.050, 0.098)
Electrolyte Imbalance	1.45 (1.02, 2.04)	0.10	0.071 (0.052, 0.098)
Sepsis	2.43 (1.51, 3.92)	0.10	0.044 (0.028, 0.069)
Urinary Tract Infection	1.53 (1.01, 2.30)	0.10	0.068 (0.046, 0.099)
**RN + LPN <1.14 HPPD**	**OR and 95% CI***	**p1**	**p2 and 95% CI**
Electrolyte Imbalance	1.40 (1.04, 1.89)	0.10	0.074 (0.056, 0.097)
Respiratory Infection	1.31 (1.01, 1.71)	0.10	0.078 (0.061, 0.099)
Sepsis	1.49 (1.02, 2.18)	0.10	0.069 (0.048, 0.098)
Urinary Tract Infection	1.60 (1.17, 2.18)	0.10	0.065 (0.048, 0.087)
**RN < 0.44 HPPD**	**OR and 95% CI***	**p1**	**p2 and 95% CI**
Electrolyte Imbalance	1.41 (1.01, 1.99)	0.10	0.073 (0.053, 0.099)
Sepsis	1.44 (1.02, 2.02)	0.10	0.072 (0.052, 0.098)
Urinary Tract Infection	1.46 (1.03, 2.06)	0.10	0.071 (0.051, 0.097)

The headings bolded in the first column represent the staff type under consideration (NA, nursing assistant; RN+LPN, licensed staff; RN, registered nurse), and condition under which the odds of hospital transfer was increased (HPPD, hours per patient day). The rest of the headings in the first column are the hospital conditions for which a statistically significant association between staffing levels and hospital transfer rates was discerned. Asterisked columns (OR, odds ratio; CI, confidence interval) reflect data taken from the CMS Report, "Appropriateness of Minimum Nurse Staffing Ratios in Nursing Homes Phase II Final Report," Chapter 2, page 17. The probability p1 is the assumed likelihood that a facility with median staffing will be in the highest decile of all facilities with respect to hospital transfer rates. The probability p2 is the calculated likelihood that a facility with recommended staffing will be in the highest decile of all facilities with respect to hospital transfer rates.

Table 6. Calculation of hospital transfer rates

**Diagnoses**	**Mean**	**Adj. mean**	**90th %ile**	**Adj. 90th %ile**	**99th %ile**	**Adj. 99th %ile**	**Adj. top 10%**	**Adj. bottom 90%**
Congestive heart failure	0.057	0.039	0.110	0.075	0.170	0.116	0.079	0.034
Electrolyte Imbalance	0.064	0.044	0.120	0.082	0.200	0.137	0.088	0.039
Respiratory Infection	0.049	0.034	0.100	0.068	0.160	0.109	0.072	0.029
Sepsis	0.020	0.014	0.050	0.034	0.110	0.075	0.038	0.011
Urinary Tract Infection	0.044	0.030	0.090	0.062	0.160	0.109	0.066	0.026
**Total**	**0.234**	**0.16**	**0.470**	**0.321**	**0.800**	**0.547**	**0.344**	**0.140**

Mean, 90th percentile, and 99th percentile hospital transfer rates derive from Chapter 2 of the CMS report to Congress, page 13. Adjusted values are downward adjusted by a factor of 1.4625 to account for the fact that the five conditions are not mutually exclusive, and that total hospital transfer rates were 16%. Adjusted top 10% values are a weighted average of the 90th and 99th percentile values. Adjusted bottom 90% values are derived from adjusted mean and adjusted top 10% values, as reported in Appendix B. Abbreviations: Adj., adjusted.

Table 7. Calculation of relative risk of hospitalization under recommended staffing

**Nurse Assistant**	**p1**	**p2 (with 95% CI)**	**Top 10%**	**Bottom 90%**	**Mean**	**Recommended (with 95% CI)**	**Optimization factor (with 95% CI)**
Congestive heart failure	0.10	0.0703 (0.0500, 0.0982)	0.079	0.034	0.039	0.038 (0.037, 0.039)	0.966 (0.943, 0.998)
Electrolyte imbalance	0.10	0.0712 (0.0517, 0.0982)	0.088	0.039	0.044	0.042 (0.041, 0.044)	0.968 (0.946, 0.998)
Sepsis	0.10	0.0437 (0.0276, 0.0685)	0.038	0.011	0.014	0.012 (0.012, 0.013)	0.887 (0.855, 0.937)
Urinary tract infection	0.10	0.0677 (0.0461, 0.0991)	0.066	0.026	0.030	0.029 (0.028, 0.030)	0.957 (0.928, 0.999)
**Licensed (RN + LPN)**	**p1**	**p2 (with 95% CI)**	**top 10%**	**bottom 90%**	**Mean**	**Recommended (with 95% CI)**	**Optimization factor (with 95% CI)**
Electrolyte imbalance	0.10	0.0735 (0.0555, 0.0965)	0.088	0.039	0.044	0.042 (0.042, 0.044)	0.971 (0.951, 0.996)
Respiratory infection	0.10	0.0782 (0.0610, 0.0991)	0.072	0.029	0.034	0.033 (0.032, 0.033)	0.972 (0.950, 0.999)
Sepsis	0.10	0.0694 (0.0485, 0.0982)	0.038	0.011	0.014	0.013 (0.012, 0.014)	0.939 (0.897,0.996)
Urinary tract infection	0.10	0.0649 (0.0485, 0.0867)	0.066	0.026	0.030	0.029 (0.028, 0.030)	0.953 (0.931,0.982)
**Registered Nurse**	**p1**	**p2 (with 95% CI)**	**top 10%**	**bottom 90%**	**Mean**	**Recommended (with 95% CI)**	**Optimization factor (with 95% CI)**
Electrolyte imbalance	0.10	0.0730 (0.0529, 0.0991)	0.088	0.039	0.044	0.042 (0.041, 0.044)	0.970 (0.948, 0.999)
Sepsis	0.10	0.0716 (0.0521, 0.0982)	0.038	0.011	0.014	0.013 (0.012, 0.014)	0.943 (0.904, 0.996)
Urinary tract infection	0.10	0.0707 (0.0512, 0.0974)	0.066	0.026	0.030	0.029 (0.028, 0.030)	0.961 (0.935, 0.996)

Values for p1 and p2 come from Appendix Table A. P1 is the probability of a median-staffed facility being in the top decile of facilities for hospital transfer rates. P2 is the probability of a facility with recommended staffing being in the top decile of hospital transfer rates. Adjusted top 10%, bottom 90%, and mean hospital transfer rates come from Appendix Table B. Whereas the median-staffed facility has a mean hospital transfer rate, the facility with recommended staffing has the hospital transfer rate under the column labeled "Recommended," which is derived as

explained in Appendix B. The optimization factor is the relative risk of hospitalization (Recommended/Mean) for a facility with recommended staffing. CI, confidence interval.
